# Immediate effects of acute Mars mission equivalent doses of SEP and GCR radiation on the murine gastrointestinal system-protective effects of curcumin-loaded nanolipoprotein particles (cNLPs)

**DOI:** 10.3389/fspas.2023.1117811

**Published:** 2023-05-05

**Authors:** Jonathan Diaz, Bradford M. Kuhlman, Nicholas P. Edenhoffer, Angela C. Evans, Kelly A. Martin, Peter Guida, Adam Rusek, Anthony Atala, Matthew A. Coleman, Paul F. Wilson, Graça Almeida-Porada, Christopher D. Porada

**Affiliations:** 1 Wake Forest Institute for Regenerative Medicine, Winston Salem, NC, United States; 2 Department of Radiation Oncology, University of California Davis School of Medicine, Sacramento, CA, United States; 3 Biosciences and Biotechnology Division, Lawrence Livermore National Laboratory, Livermore, CA, United States; 4 NASA Space Radiation Laboratory, Brookhaven National Laboratory, Upton, NY, United States; 5 Biological Sciences Division, Pacific Northwest National Laboratory, Richland, WA, United States

**Keywords:** space radiation, HZE exposure, gastrointestinal, DNA damage, apoptosis, H2AX foci, curcumin, countermeasure

## Abstract

**Introduction:**

Missions beyond low Earth orbit (LEO) will expose astronauts to ionizing radiation (IR) in the form of solar energetic particles (SEP) and galactic cosmic rays (GCR) including high atomic number and energy (HZE) nuclei. The gastrointestinal (GI) system is documented to be highly radiosensitive with even relatively low dose IR exposures capable of inducing mucosal lesions and disrupting epithelial barrier function. IR is also an established risk factor for colorectal cancer (CRC) with several studies examining long-term GI effects of SEP/GCR exposure using tumor-prone APC mouse models. Studies of acute short-term effects of modeled space radiation exposures in wildtype mouse models are more limited and necessary to better define charged particle-induced GI pathologies and test novel medical countermeasures (MCMs) to promote astronaut safety.

**Methods:**

In this study, we performed ground-based studies where male and female C57BL/6J mice were exposed to γ-rays, 50 MeV protons, or 1 GeV/n Fe-56 ions at the NASA Space Radiation Laboratory (NSRL) with histology and immunohistochemistry endpoints measured in the first 24 h post-irradiation to define immediate SEP/GCR-induced GI alterations.

**Results:**

Our data show that unlike matched γ-ray controls, acute exposures to protons and iron ions disrupts intestinal function and induces mucosal lesions, vascular congestion, epithelial barrier breakdown, and marked enlargement of mucosa-associated lymphoid tissue. We also measured kinetics of DNA double-strand break (DSB) repair using gamma-H2AX- specific antibodies and apoptosis via TUNEL labeling, noting the induction and disappearance of extranuclear cytoplasmic DNA marked by gamma-H2AX only in the charged particle-irradiated samples. We show that 18 h pre-treatment with curcumin-loaded nanolipoprotein particles (cNLPs) delivered via IV injection reduces DSB-associated foci levels and apoptosis and restore crypt villi lengths.

**Discussion:**

These data improve our understanding of physiological alterations in the GI tract immediately following exposures to modeled space radiations and demonstrates effectiveness of a promising space radiation MCM.

## Introduction

During future missions beyond low Earth orbit (LEO), such as those planned to the Moon, near-Earth asteroids, and Mars, astronauts will face poorly defined health risks as a result of exposure to the complex space ionizing radiation (IR) environment consisting of solar energetic particles (SEP) including protons and light ions and galactic cosmic rays (GCR) ranging from high-energy protons to high atomic number and energy (HZE) charged particles, often referred to as heavy ions. Although heavy ion fluences are orders of magnitude lower than proton and He-4 ions, their associated high linear energy transfer (LET) and correspondingly higher relative biological effectiveness (RBE) values results in them delivering a large portion of the overall dose equivalent that an astronaut will receive during an extended mission. SEP/GCR exposures are chronic, low dose-rate (LDR) on the order of 0.5–1 mGy/day in deep space and ~0.2–0.4 mGy/day on the surfaces of Mars and the Moon with additional dose contributions on these surfaces from albedo neutrons, gamma rays, and other charged particle types (Durante and Cucinotta, 2011; Zeitlin et al., 2013; Hassler et al., 2014; Zhang et al., 2020). Sporadic solar particle events (SPEs) however present a relatively high dose-rate (HDR) exposure scenario whereby an astronaut could potentially receive a dose of ~0.5–1 Gy within minutes to hours capable of generating symptoms of gastrointestinal (GI) syndrome-associated acute radiation sickness (ARS) (Townsend, 2005; Kim et al., 2009; Townsend et al., 2018). Long-duration spaceflight can result in the accumulation of IR exposures that may reach or exceed NASA career exposure limits (600 mSv weighted dose equivalent)—potentially producing both short- and long-term deleterious effects on human physiological systems that may limit mission success and increase risks of central nervous system (CNS) disruption, cardiovascular disease (CVD), and cancer morbidity/mortality in astronauts (Cucinotta et al., 2001; Townsend, 2005; Cucinotta and Durante, 2006; Durante and Cucinotta, 2008; Rodman et al., 2017; Almeida-Porada et al., 2018; Onorato et al., 2020; Simonsen et al., 2020). Physical, biological, and pharmacological-based radiation protection and mitigation strategies will be required to ensure successful mission outcomes, complete with charged particle-specific radiation medical countermeasures (MCMs) that are easily administered, can safely be stored long-term and maintain efficacy, and show beneficial effects in multiple radiosensitive tissue compartments, e.g., the bone marrow (BM) niche and gastrointestinal (GI) tract (Bokhari et al., 2022; DiCarlo et al., 2022).

With research being conducted in this area at ground-based charged particle accelerator facilities and aboard the International Space Station (ISS), our understanding of the biological effects resulting from exposures to this unique/complex radiation environment is still incomplete and complicated by the paucity of human epidemiological studies for these more exotic radiation types. This makes it difficult to accurately extrapolate disease risks from terrestrial radiation exposures from low linear energy transfer (LET) photon radiations and high LET alpha particles from inhaled radon/radon progeny. Accelerated HZE ions can have orders of magnitude higher LET values compared to photons and low dose exposures (≤50 cGy) of both low LET protons and intermediate to high LET heavy ions have been shown to directly induce complex, difficult to repair clusters of double-strand breaks (DSB) and other DNA lesion types in highly-localized DNA regions (Sutherland et al., 2000; Sutherland et al., 2001; Saha et al., 2014; Bennett et al., 2022) whose proper repair is exacerbated in microgravity conditions (Moreno-Villanueva et al., 2017; Hada et al., 2018; Low et al., 2018; Yamanouchi et al., 2020; Beheshti et al., 2021).

It is well documented that the GI system constitutes one of the most radiosensitive tissues of the body, and sensitive to the direct (DNA damaging) and indirect (inflammation-associated) effects of IR exposures. This is due to high turnover rates of the intestinal epithelium, a process fueled by intestinal stem cell (ISC) division and differentiation originating from the base of intestinal crypts up to postmitotic cells at the upper villi positions (Clevers, 2013). Low dose IR exposures have been shown to induce crypt cell apoptosis and lead to mucosal lesions and loss of epithelial barrier function, which increases mucosal permeability, nutrient and fluid loss, and gut pathogen infiltration. The net effect of these insults is the establishment of a state of chronic mucosal inflammation resulting in tissue loss and fibrosis (Shadad et al., 2013; Takiishi et al., 2017; Chen et al., 2021). IR is an established risk factor for colorectal cancer (CRC) (Thompson et al., 1994; Pawel et al., 2008; Grant et al., 2017) and studies by the Fornace group demonstrated acute HDR exposures to accelerated protons and Fe-56 ions delivered at the NASA Space Radiation Laboratory (NSRL) resulted in long-term GI effects in wildtype C57BL/6J mice and significantly enhanced the development and progression of intestinal tumors in mice harboring mutations in the Apc gene (Trani et al., 2010; Datta et al., 2013; Trani et al., 2014; Kumar et al., 2018; Kumar et al., 2019; Suman et al., 2019; Suman et al., 2020; Suman et al., 2021). Fewer studies have investigated immediate short-term (≤24–48 h post-irradiation) acute GI effects following low dose charged particle irradiation using these mouse models. A study by Purgason et al. in BALB/c mice demonstrated decreased crypt cell proliferation, survival, and mucosal surface area along with the induction of apoptosis and apoptosis-related gene expression for whole-body 250 MeV proton doses as low as 10 cGy (Purgason et al., 2018).

In this report, we performed ground-based studies at the NSRL with male and female 5–6-week-old C57BL/6J mice to better define GI responses immediately (≤24 h) following exposures to doses and ion species typical of the space radiation environment. These experiments were designed to serve as an *in vivo* analog to a series of *in vitro* experiments employing a human “gut-on-a-chip” microfluidic physiological systems (MPS) platform (Shah et al., 2016) used by our co-investigators (manuscript in preparation). We also tested the ability of intravenously delivered curcumin-laden nanolipoprotein particles (cNLPs) that we recently developed (Evans et al., 2022) as a potential medical countermeasure (MCM) to radioprotect the GI tract from the damaging effects of charged particle radiations. A wealth of literature supports curcumin’s ability to modulate multiple IR- and cancer-associated pathways involved via its potent antioxidant properties, but its poor solubility and bioavailability has limited its therapeutic use (Weiss and Landauer, 2003; Akpolat et al., 2009; Wang et al., 2017; Kostova et al., 2021). We recently reported that packaging of curcumin in biomimetic nanolipoprotein particles (NLPs) greatly increases its solubility and stability, and pre- or post-IR treatment of human AG05965/MRC-5 fibroblasts with 27 μM cNLPs modulates the expression of multiple genes that regulate DNA repair including CDKN1A and BCL2, and confers both radioprotection and radiomitigation in non-cycling quiescent cultures (Evans et al., 2022). Unfortunately funding and NSRL facility access/cost limitations prevented us from doing longer-term evaluations of low dose-exposed mouse cohorts as well as investigating potential dose-rate effects to better mimic the chronic dose-rates encountered in deep space. The exposures we conducted are consistent with acute doses and dose-rates experienced during sporadic solar particle events (SPE) that can deliver 0.5–1 Gy doses of lower-energy protons and light ions (<100 MeV/n) within minutes to hours. Our results show that exposures to 50 MeV protons and 1 GeV/n Fe-56 ions induces marked structural and functional alterations, DSB-associated foci including extranuclear cytoplasmic DNA fragments marked with gamma-H2AX-specific antibodies, and apoptosis measured by TUNEL staining within intestinal sections recovered and processed from the exposed mice at 15 min to 24 h timepoints. Importantly, we also demonstrate that DSBs and apoptosis induction are reduced in mice pretreated for 18 h with cNLPs delivered intravenously by tail vein injection, with partial restoration of epithelial barrier length, providing evidence for *in vivo* radioprotection by this space radiation MCM candidate. It is our hope that these results will assist NASA in its efforts in space radiation risk assessment modeling and provide a safe nutraceutical-based MCM to promote astronaut safety during extended deep space missions.

## Materials and methods

### Mice husbandry and irradiations, dosimetry, and harvesting of the small intestine

All animal procedures were performed under protocols approved by the Institutional Animal Care and Use Committees (IACUC) at both Wake Forest University Health Sciences (WFUHS protocol A15-137) and Brookhaven National Laboratory (BNL protocol 500) following the Guide for the Care and Use of Laboratory Animals prepared by the Institute of Laboratory Animal Resources, National Research Council, and U.S. National Academy of Sciences. For this study, we exposed a total of 108 wildtype C57BL/6J mice (male and female) aged 5–6 weeks to the following IR types and doses at the NSRL and using the BNL Biology Department’s J.L. Shepherd Mark I Model 68A cesium-137 cabinet irradiator:

1. Sham (0 cGy unirradiated control); n = 24
2. 100 cGy of 662 keV Cs-137 γ-rays (LET = 0.91 keV/μm); n = 24
3. 100 cGy of 50 MeV protons (LET = 1.26 keV/μm); n = 24
4. 50 cGy of 1 GeV/n Fe-56 ions (LET = 151.49 keV/μm); n = 36

**FIGURE 1:**
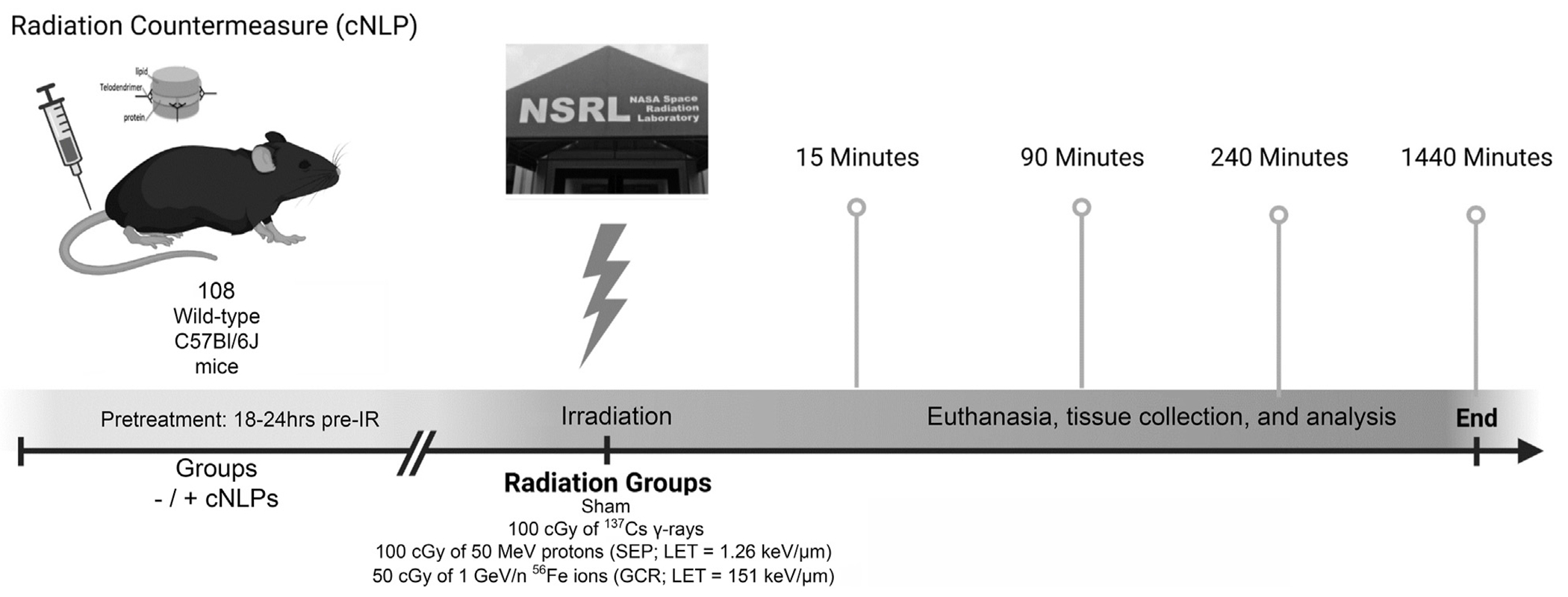
Diagrammatic representation of overall experimental design showing IR exposure schemes, pretreatment with cNLP GI MCM (+/−), and euthanasia/tissue collection times post-IR at which mice from each IR cohort were euthanized for analyses. *n* = 108 total C57BL/6J mice; specific IR exposure groups were as follows: sham (0 cGy control) *n* = 24 (18 without cNLPs, 6 with cNLPs); cesium-137 γ-rays (photon reference control) *n* = 24 (18 without cNLPs, 6 with cNLPs); 50 MeV protons (model SEP radiation) *n* = 24 (18 without cNLPs, 6 with cNLPs); 1 GeV/n Fe-56 ions (model GCR radiation) *n* = 36 (24 without cNLPs, 12 with cNLPs).

Mice were purchased from Jackson Laboratories (Bar Harbor, ME, United States) and shipped directly to the Brookhaven National Laboratory Animal Facility (BLAF) in NY approximately 1 week prior to scheduled beam times to acclimate to the facility. Mice were housed communally 3–6 mice per cage under a 12-h light/dark cycle with standard mouse chow and filtered water provided *ad libitum*. On the mornings of scheduled beam times, all mice (those to be irradiated and the sham controls) were transported by BLAF personnel to the NSRL animal facility. Mice to be irradiated were loaded into individual clean Lucite mouse holders/boxes (3 × 1.5 × 1.5 inches) with pre-drilled air holes and placed in custom 6-box support frames in the NSRL animal facility minutes prior to scheduled beam time, transported into the target room, and exposed (6 mice at a time in a single 6-box support) unanesthetized at room temperature on the NSRL beamline (≤1–2 min exposure times) after which they were immediately returned to their cages. To control for effects due to handling and/or stress, sham controls were also loaded into Lucite holders for 3 min and were then returned to their cages. To avoid any potential confounding effects caused by residual scattered radiation present in the target room, sham controls were kept in the animal housing room at NSRL, rather than performing “walk-in walk-out” controls by bringing them into the target room. Accelerated 50 MeV proton and 1 GeV/n Fe-56 ion doses were verified by the NSRL physics/dosimetry team using calibrated NIST-traceable ion chambers located upstream and downstream of the target position. Dosimetry of these NSRL mouse irradiations was modeled using the “Digimouse” digital mouse phantom and associated dosimetry calculations provided by Simonsen et al. (Simonsen et al., 2020) with delivered tissue doses within 6% of the prescribed doses. Dose rates ranged from 70.8 to 75.5 cGy/min and 49–50 cGy/min for the proton and iron ion irradiations respectively. Cesium-137 662 keV γ-ray irradiations were conducted as photon reference controls with un-anesthetized mice loaded into clean NSRL mouse holders with pre-drilled air holes and placed in a custom 6-box foam support frame at position #2 perpendicular to the source to ensure uniform dose distribution and irradiated with 100 cGy at a dose-rate of 140.85 cGy/min using the BNL Biology Department’s J. L. Shepherd Mark I Model 68A cesium-137 cabinet irradiator. LET values for the IR types are: 1.26 keV/μm (50 MeV protons), 151.49 keV/μm (1 GeV/n Fe-56 ions), and 0.91 keV/μm (Cs-137 γ-ray-induced photoelectrons (ICRP, 2003)); dose to particle fluence conversions were calculated according to (Schardt et al., 2010). Cohorts from each IR group and corresponding sham controls were euthanized by CO2 asphyxiation and cervical dislocation in either the NSRL or BLAF animal facilities at 15 min, 90 min, 4 h, and 24 h post-irradiation with the GI tract removed and fixed for subsequent immunohistochemical/immunocytochemical processing and staining (see below). Figure 1 shows an overview of the study design.

### Curcumin nanolipoprotein particles

18–24 h prior to irradiation, cohorts of C57BL/6J mice from each IR group [6 mice (3M/3F) each from the n = 24 sham, gamma and proton groups and 12 mice (6M/6F) from the n = 36 iron ion groups] were injected with 27 μM (10 μg/mL) curcumin nanolipoprotein particles (cNLPs) in sterile Dulbecco’s phosphate-buffered saline (D-PBS) via intravenous (IV) tail vein injection to test its ability to serve as a GI-specific space radiation MCM. Development, production, and characterization of these cNLPs as an *in vitro* radioprotector and radiomitigator in human cells is documented in (Evans et al., 2022). Briefly, cNLP solutions were prepared by resolubilizing lyophilized cNLPs prepared per (Evans et al., 2022) at the Lawrence Livermore National Laboratory (LLNL) in sterile water and subsequently diluting it 1:1 with PBS to 0.1 mg/mL with 100 μL injected into the tail vein under a warming lamp in the BLAF.

### Histology, immunofluorescence, and immunohistochemistry

The duodenum of the small intestine was harvested from each mouse at the time of euthanasia (15 min, 90 min, 4 h, and 24 h post-irradiation) and prepared using a standard “Swiss-rolling” technique. Briefly, tissues were gently flushed with ice-cold PBS and fixed overnight in 10% neutral-buffered formalin (Leica Biosystems; 3,800,598). Following fixation, tissues were cut longitudinally, rolled, and placed into tissue cassettes for processing and paraffin embedding. 5-μm tissue sections were prepared via microtome and deposited on positively charged Superfrost^®^ Plus Micro Slides (VWR, Radnor, PA). Slides were then deparaffinized and rehydrated and stained with hematoxylin and eosin using Leica ST5010 Autostainer XL automated processor or antigen retrieval was performed using either citrate buffer (eBioscience^™^ IHC Antigen Retrieval Solution—Low pH; Invitrogen/ThermoFisher Scientific, Waltham, MA; 00-4955-58) or EDTA buffer (eBioscience^™^ IHC Antigen Retrieval Solution—High pH; Invitrogen; 00-4956-58) per antigen of interest.

Tight junction length and integrity were assessed following EDTA antigen retrieval using a rabbit primary polyclonal antibody against Claudin-3 (Abcam; ab15102; 1:150 dilution) and AlexaFluor594-conjugated goat anti-rabbit IgG (H + L) secondary antibody (Invitrogen; A-11037; 1:500 dilution). Intestinal stem cells were stained following citrate antigen retrieval and permeabilization with 0.2% Triton X-100 for 5 min. Rat primary monoclonal antibody (Invitrogen; 14-9896-82; 1:200) against the ISC marker Musashi-1 and a rabbit primary polyclonal antibody against PCNA (Abcam; ab18197; 1:1000) were used, detected with Alexa Fluor^®^ 488-conjugated donkey anti-goat IgG (H + L) (Abcam, ab150129) and Alexa Fluor^®^ 594-conjugated goat anti-rabbit IgG secondary antibody secondary antibodies (Invitrogen/ThermoFisher Scientific; A-11037; 1:500 dilution) respectively. Sections were counterstained with 0.2-μg/mL DAPI (ThermoFisher Scientific; 62,248) and cover-slipped using ProLong Gold Antifade Reagent (ThermoFisher Scientific; P10144).

Duodenal samples were also processed at each time point post-IR for analysis of apoptosis via the terminal deoxynucleotidyl dUTP nick end-labeling (TUNEL) assay following the manufacturer’s instructions (Abcam; TUNEL Assay Kit HRP-DAB, ab206386). Each specimen was then counterstained with methyl green for 2 min and coverslipped with Permount (ThermoFisher Scientific). Sections from each duodenum were also subjected to immunofluorescence (IF) analysis using a rabbit primary monoclonal antibody to γ-H2AX pS139 (Abcam; ab81229; 1: 100 dilution) and Alexa Fluor^®^ 488-conjugated goat anti-rabbit IgG (H + L) secondary antibody (Invitrogen/ThermoFisher Scientific, A-11034; 1:500 dilution) to establish levels and repair kinetics of DNA double-strand break (DSB)-associated nuclear foci induced by IR exposure. In brief, sections were permeabilized in Tris-buffered saline containing 0.2% Triton X-100 (TBS-T) followed by 24-h incubation at 4°C with the primary antibody. After 2 h of incubation at RT with the secondary antibody, sections were washed twice in TBS, counterstained with DAPI, and coverslipped using ProLong Gold Antifade Reagent (ThermoFisher Scientific; P10144). For all immunofluorescence assays, following permeabilization, each tissue section was treated with an autofluorescence quencher solution (TrueBlack^®^ Lipofuscin, 23,007) to diminish background autofluorescence present in murine intestinal tissues. All morphological changes were assessed using brightfield microscopy (Leica DM4000B), and fluorescence imaging was performed on an Olympus FV1200 Spectral Laser scanning Confocal Microscope.

### Histopathology analysis

Investigators who were blinded to sample identity/treatment group examined all H&E-stained tissue samples to assess GI tissue damage resulting from exposure to each IR dose/type. ImageJ software packages (NIH) were used to perform analysis of the images in this study. Throughout this manuscript, we used ImageJ to create specific macros with multiple functions to automate tasks including normalizing, processing, and analyzing large numbers of images. This included a normalizing step utilizing color deconvolution to separate images into component color channels, and tasks like sharpening and contrast leveling. The macro also included the “Subtract Background” function to isolate and analyze features in images above a uniform background intensity. After background removal, we used auto-thresholding algorithms, a common technique in image processing and analysis, to segment images into different regions based on pixel intensity values. The “Watershed” threshold segmentation algorithm function was used to separate touching or overlapping objects in the images by tracing the boundaries of objects and after normalizing, we used the “Analyze Particles” function to identify and measure features in the image. In some cases, it was necessary for us to fine-tune the image analysis parameters to obtain accurate results by “training” each dataset. We ran the macro multiple times, adjusting parameters as needed to improve overall performance and visually inspecting the results to ensure accuracy. All readings obtained via these custom macros were processed via Excel spreadsheets and were then imported into RStudio using the readxl package. The following GitHub link contains ImageJ algorithms and R scripts that were used for the analyses detailed in this manuscript: https://github.com/jhd8593/ImageJ_IHC_Java.git

This approach enabled us to obtain unbiased quantitation of apoptotic TUNEL labeling, γ-H2AX foci, and Claudin-3-positive epithelial tight junction spacing within the small intestine of irradiated mice and unirradiated controls. Plots and statistical analysis were performed using RStudio. ANOVA analyses were used to determine significant differences between groups, and we performed pairwise t-tests with a Bonferroni correction to determine specific differences between group means. To assess “normality” of the data, we performed a Shapiro-Wilk test. For all datasets with sufficient “n” to yield a meaningful result via Shapiro-Wilk test (as this test is highly sensitive to sample size), we obtained *p*-values higher than 0.05, indicating that the null hypothesis cannot be rejected, and the data thus appear to be normally distributed.

## Results

### Histopathological findings after ionizing radiation exposure

**FIGURE 2:**
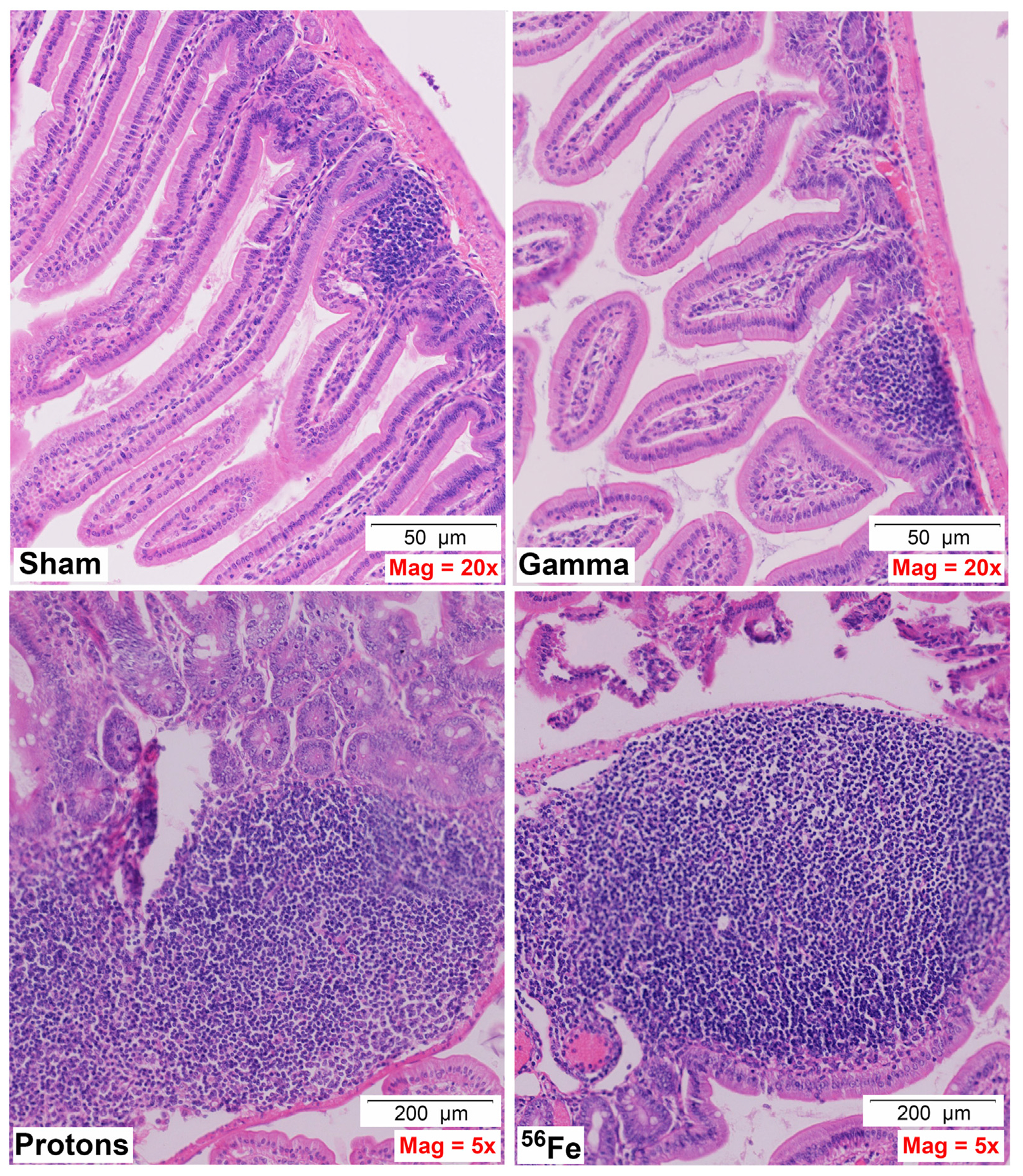
Representative H&E-stained sections of duodenal samples collected from C57BL/6J mice exposed to each IR scheme illustrating IR-associated swelling of lymph nodes/MALT (mucosa-associated lymphoid tissue)/Peyer’s patches–note magnification for γ-ray exposure is ×20 to allow better visualization of the lymph node, while magnification for protons and Fe-56 ions is ×5 (50- or 200-μm scale bars, as marked).

**FIGURE 3:**
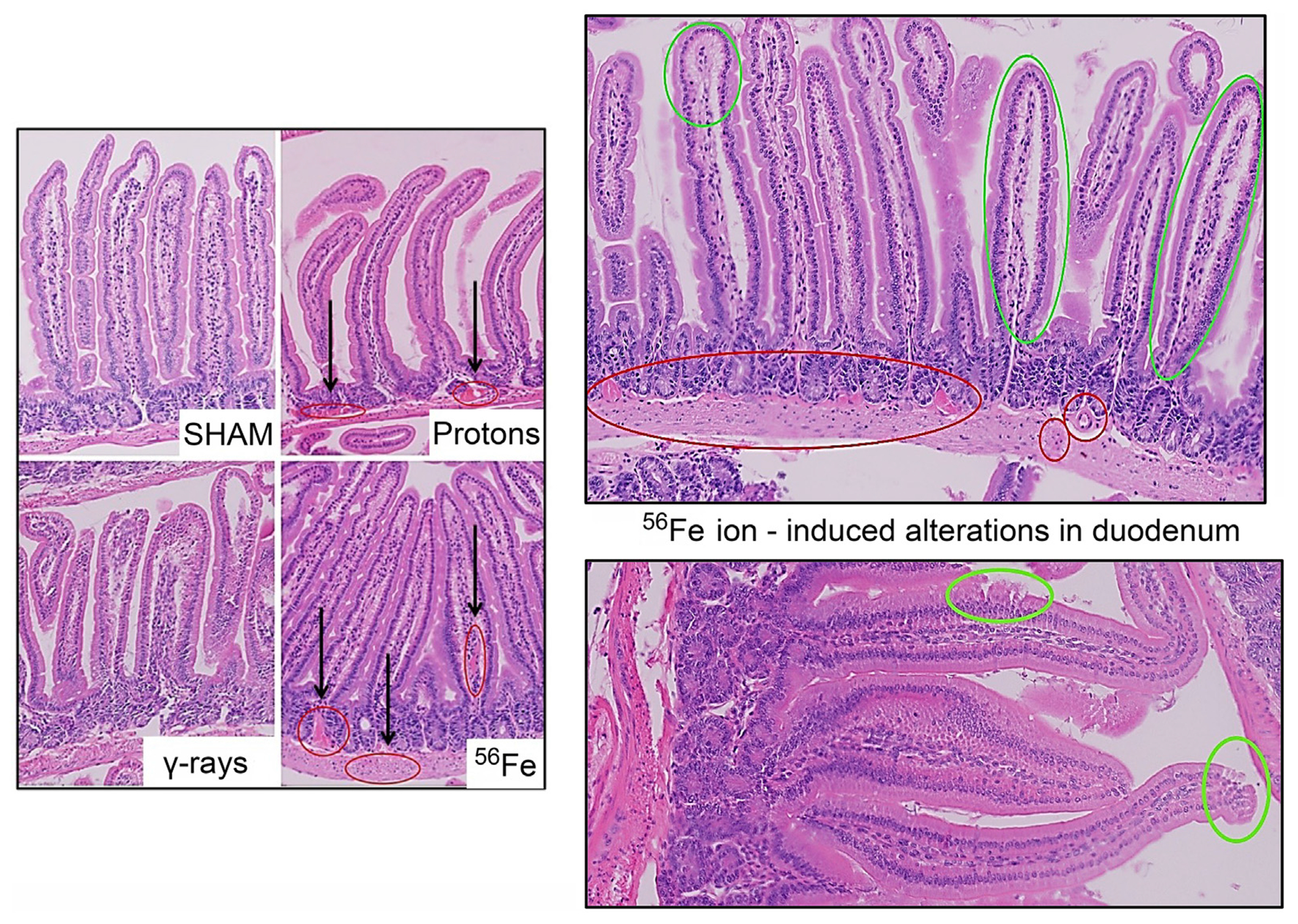
Representative H&E-stained sections of duodenal samples collected from C57BL/6J mice exposed to each IR scheme illustrating IR-associated vascular damage/abnormalities. Vascular congestion, dilation, and possible bleeding (red circles); and possible edema and epithelial disruption (green circles) in the duodenum of a mouse exposed to Fe-56 ions.

Acute whole-body irradiation with 100 cGy of 50 MeV protons or 50 cGy of 1 GeV/n Fe-56 ions both induced marked swelling/enlargement of local lymph nodes in the duodenum of mice exposed to each of these ions by 24 h post-IR. Such swelling was not observed in either sham-irradiated or 100 cGy γ-ray-irradiated groups. Images of enlarged mucosa-associated lymphoid tissue from representative animals in each IR group are shown in Figure 2. While there was no evidence of mucosal ulcers, inflammation, or crypt epithelial degeneration at these immediate early timepoints post-IR, multiple structural alterations were seen in the animals exposed to the two charged particle types that were not present in the sham-irradiated or γ-ray groups, including vascular congestion (noted in red circles) and edema (noted in green circles) within the villi (Figure 3), as well as disruption of the lymphatics, specifically the lacteal, and occasional splitting of crypts. In addition, focal infiltration of neutrophils/granulocytes was observed in the lymph nodes of three of the mice exposed to Fe-56 ions but not in the other groups. However, as these alterations can occur on rare occasions in sentinel mice housed in animal facilities due to undetected infectious agents and were not present in all animals in each IR group, these findings when considered groupwise were not deemed to be “pathologically important” (although it is interesting that they only occurred in the charged particle-irradiated cohorts).

### Epithelial barrier thinning and disruption

**FIGURE 4:**
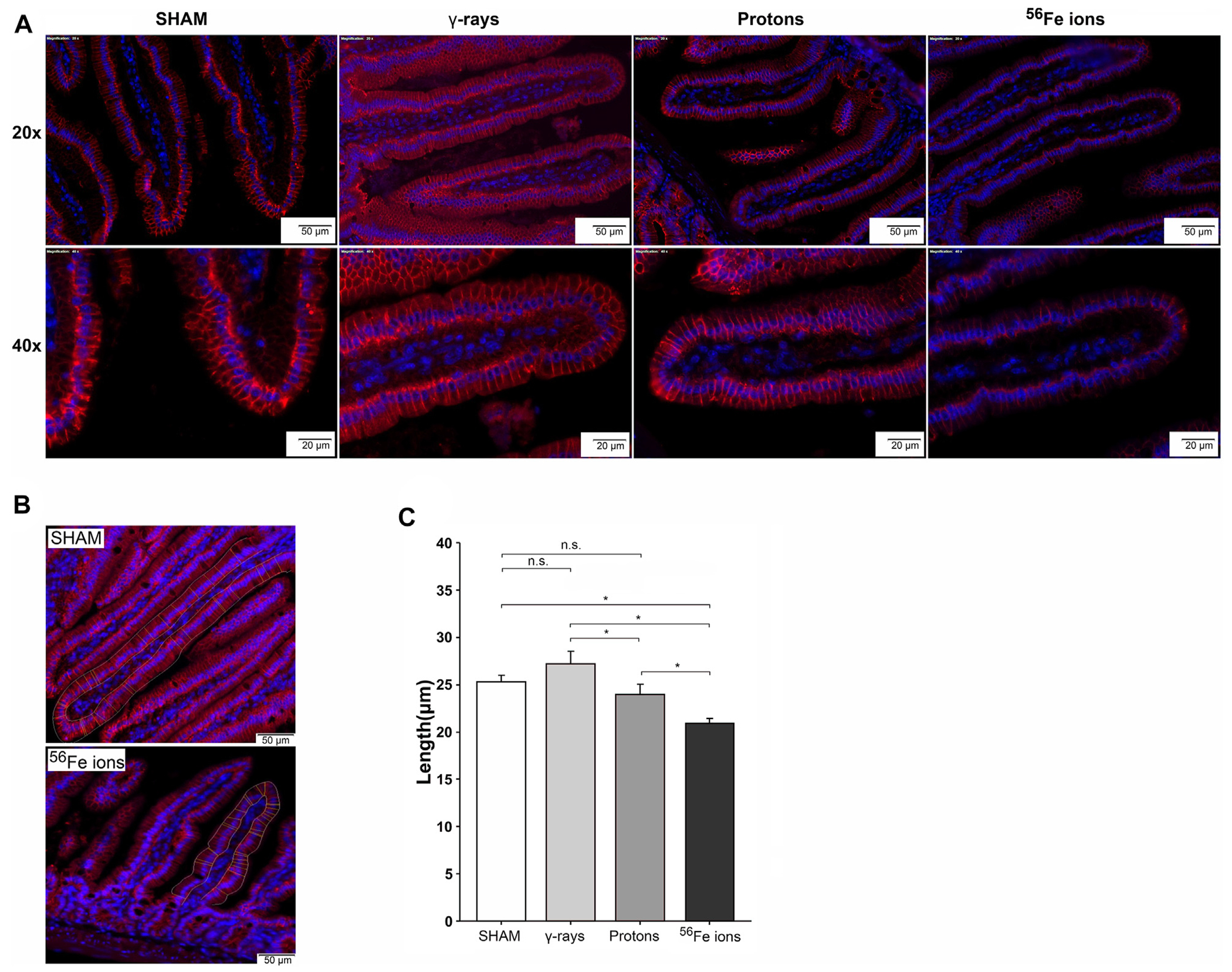
Immunofluorescence analysis of tight junction-associated Claudin-3 staining as a measure of epithelial barrier integrity. Panel **(A)** Representative confocal images of duodenal samples from mice exposed to sham (0 cGy), 100 cGy of cesium-137 γ-rays, 100 cGy of 50 MeV protons, or 50 cGy of 1 GeV/n Fe-56 ions stained with an antibody specific to Claudin-3 (red). Top panel shows ×20 magnification (50-μm scale bar); bottom panel shows ×40 magnification (20-μm scale bar). Panel **(B)** Close-up images of Claudin-3 staining in the duodenum of a sham-irradiated (top) and Fe-56 ion-irradiated (bottom) mouse at 24 h post-IR (50-μm scale bar). **(C)** Tight junction length quantified using a custom ImageJ script written to measure the length of the Claudin-3-positive barrier between adjacent epithelial cells. Multiple “regions of interest” (ROIs) were analyzed in each section, and the script computed an average of 30 random measurements (in μm) of the narrow junction (*n* = 3 ROIs per section); *p < 0.05.

To determine modeled SEP/GCR radiation-induced effects on epithelial barrier functionality in the small intestine, we performed immunohistochemical staining with an antibody specific to Claudin-3, a tight junction-associated protein within villi epithelial cells that plays a critical role in the formation and maintenance of the barrier that separates cells of the gut from the intestinal lumen (Van Itallie and Anderson, 2006; Shim et al., 2015). Visual inspection of Claudin-3 staining intensity reveals that proton exposure led to mild disturbance of barrier function, while Fe-56 ion irradiation causes more disruption (Figure 4A). To obtain quantitative data regarding barrier function, we developed and utilized a custom ImageJ script to analyze the immunofluorescence images, measuring the tight junctions of each villus with automated two-user polylines (Figure 4B). Multiple “regions of interest” (ROIs) were analyzed per section, and the script computed an average of 30 random μm-based measurements of the narrow junction (n = 3 ROIs per section). This unbiased quantitative approach confirmed our prior visual observations of Claudin-3 staining intensity that the tight epithelial junctions appeared to be reduced in length in duodenum samples of mice exposed to protons and especially in those animals exposed to Fe-56 ions as compared to sham-irradiated or γ-ray controls (Figure 4C).

### Effects of SEP/GCR on the intestinal stem cell pool

**FIGURE 5:**
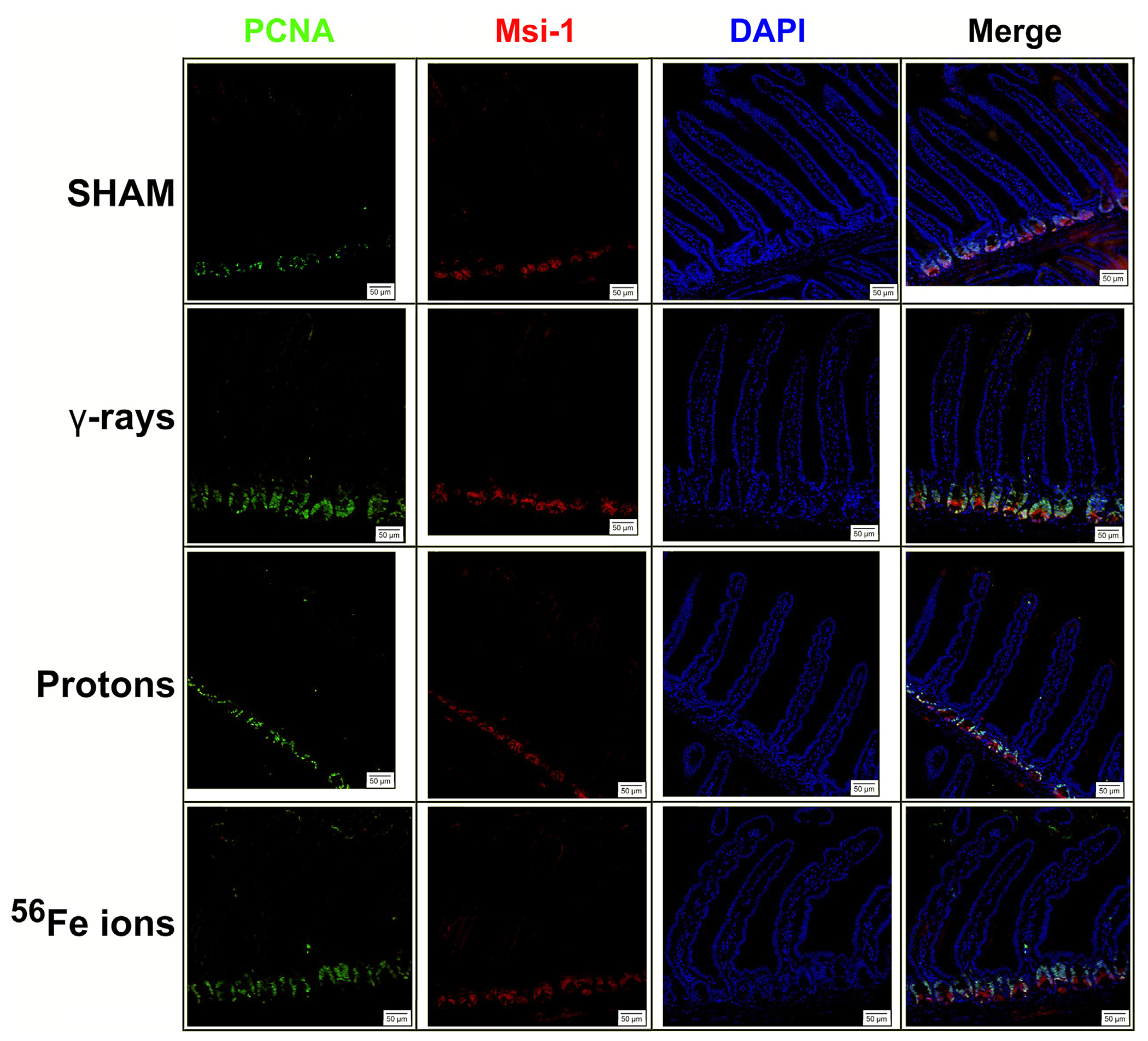
Immunohistochemical staining for Musashi-1 (red) and proliferating cell nuclear antigen (PCNA; green) of duodenal sections from mice exposed to various IR schemes collected at 24 h post-irradiation to assess Msi-1-positive intestinal stem cell (ISC) and PCNA-positive proliferating cell numbers and locations. Representative images at ×20 magnification (50-μm scale bar).

**FIGURE 6:**
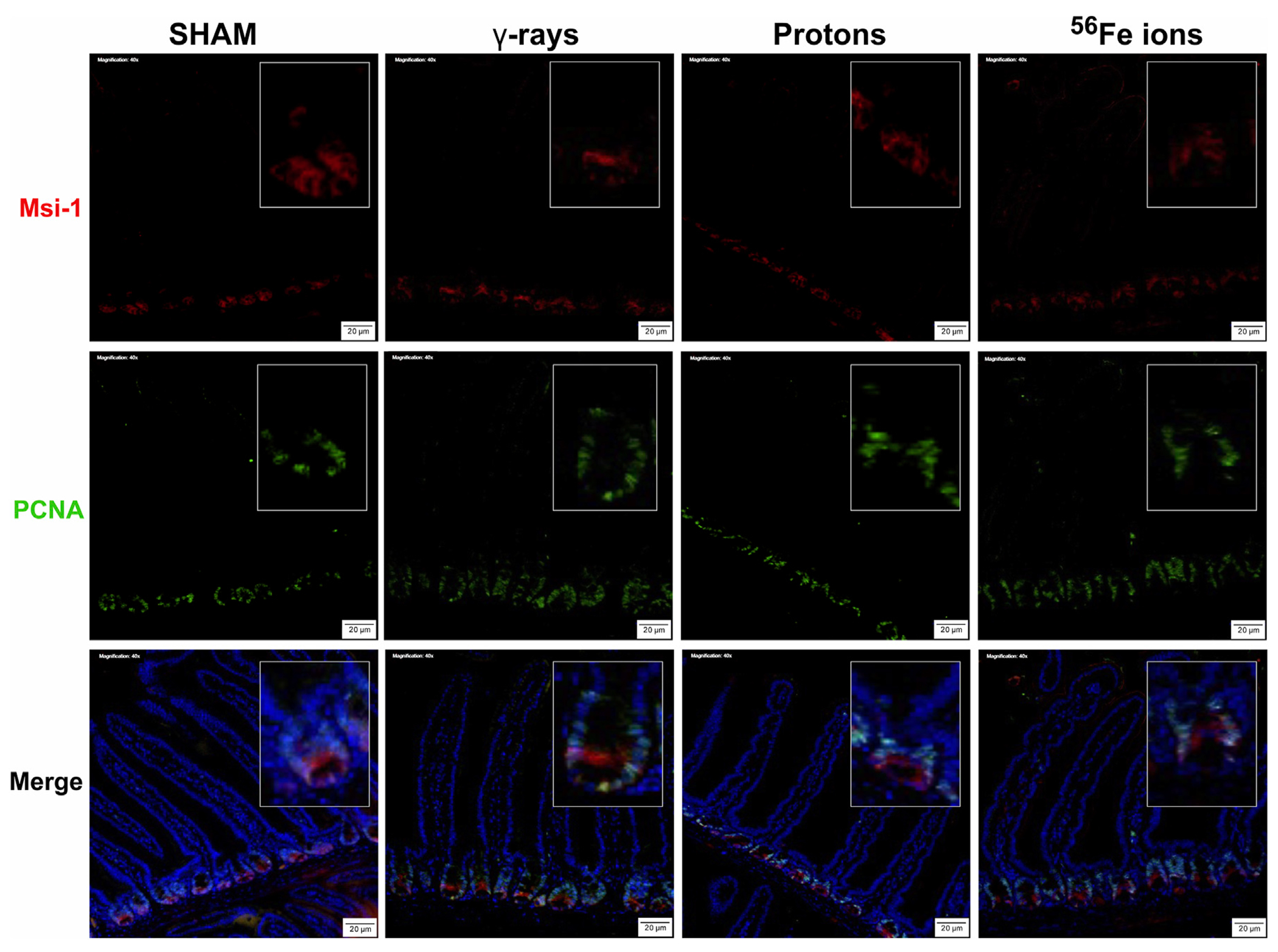
Immunohistochemical staining for Musashi-1 (red) and PCNA (green) of duodenal sections from mice exposed to various IR schemes collected at 24 h post-irradiation to assess Msi-1-positive ISC and PCNA-positive proliferating cell numbers and locations. Representative images at ×40 magnification (insets at ×2.5 additional magnification, 20-μm scale bars).

**FIGURE 7:**
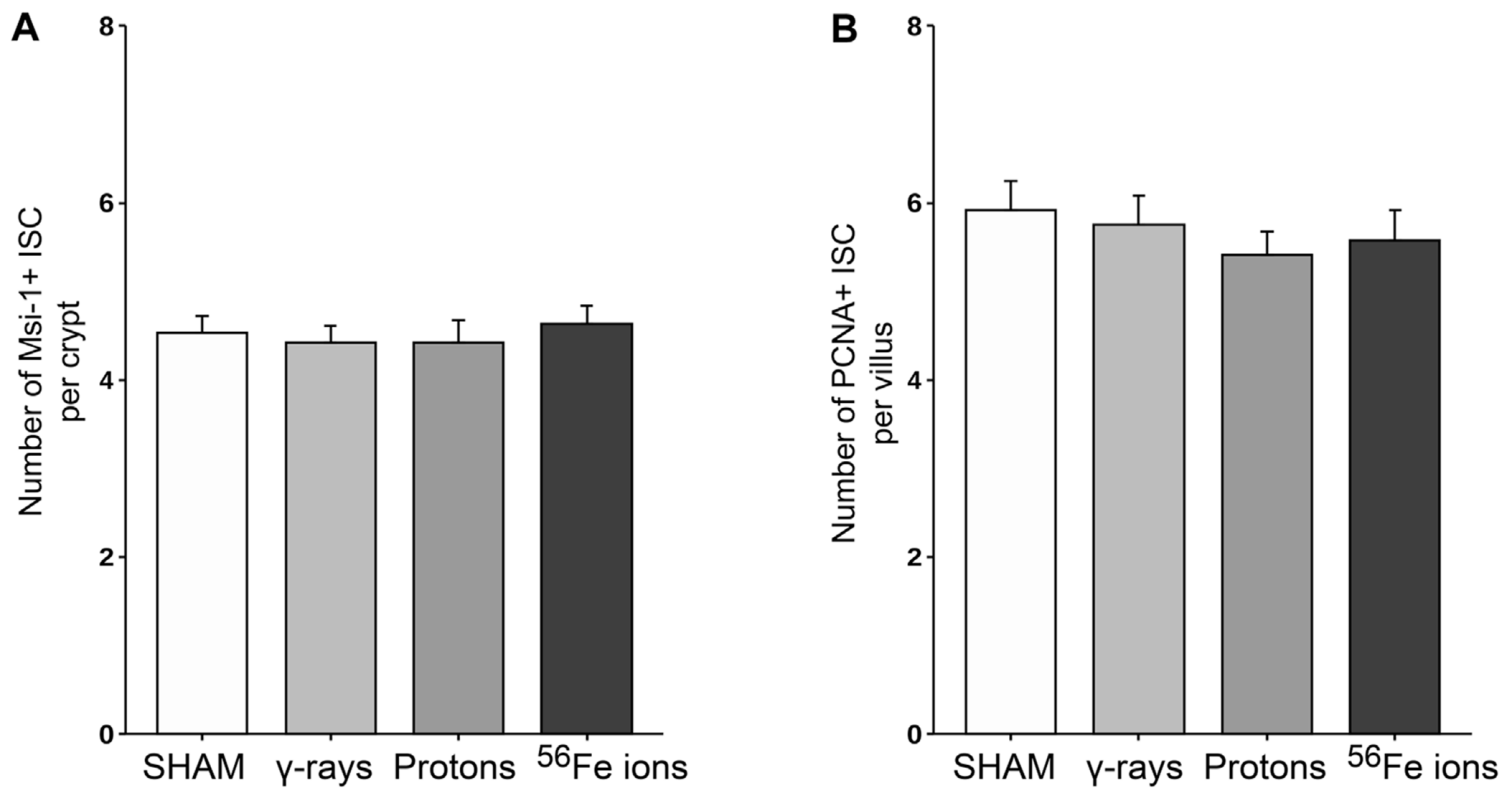
Quantification of ISC and proliferating cells within duodenal sections from mice exposed to various IR schemes collected at 24 h post-irradiation, using a custom ImageJ script. Panel **(A)** Plot of Msi-1-positive ISC per crypt quantitated using a custom ImageJ script (*n* = 3 ROIs per section). Panel **(B)** Plot of PCNA-positive (proliferating) plus Msi-1-positive ISC per crypt quantitated using a custom ImageJ script (*n* = 3 ROIs per section).

The epithelium of the small intestine is continually replaced by intestinal stem cells (ISC) that reside within crypts at the base of each villus through carefully orchestrated processes of migration and differentiation. As such, IR-induced killing of ISC cells through accompanying mitotic cell death and apoptosis mechanisms can exert a pronounced deleterious effect on gut physiology and function by disrupting villi functioning and replenishment. To examine whether this occurred following proton or iron ion irradiation, we next measured how exposures affected the number, position, and cycling status of ISC within the mouse duodenum within 24 h post-irradiation using immunofluorescence-based analyses using antibodies specific to Musashi-1 (Msi-1), a marker of ISC (Potten et al., 2003), and proliferating cell nuclear antigen (PCNA), a marker of actively cycling cells. All 3 IR types led to an increase in the number of Msi-1-positive (red) and PCNA-positive (green) proliferating cells within and around the crypts of the villi (Figures 5, 6), although these changes were not statistically significant (*p* ≤ 0.05; Figure 7). Performing ImageJ-based quantitative analyses on these sections also enabled us to assess whether the exposures affected the proliferation status of ISC within the crypts by quantifying the number of Msi-1-positive cells that were likewise positive for PCNA. As seen in Figure 7, the charged particle exposures showed a trend towards reduced numbers of proliferating ISC per crypt, but again these differences did not achieve statistical significance.

### IR-induced programmed cell death (apoptosis)

**FIGURE 8:**
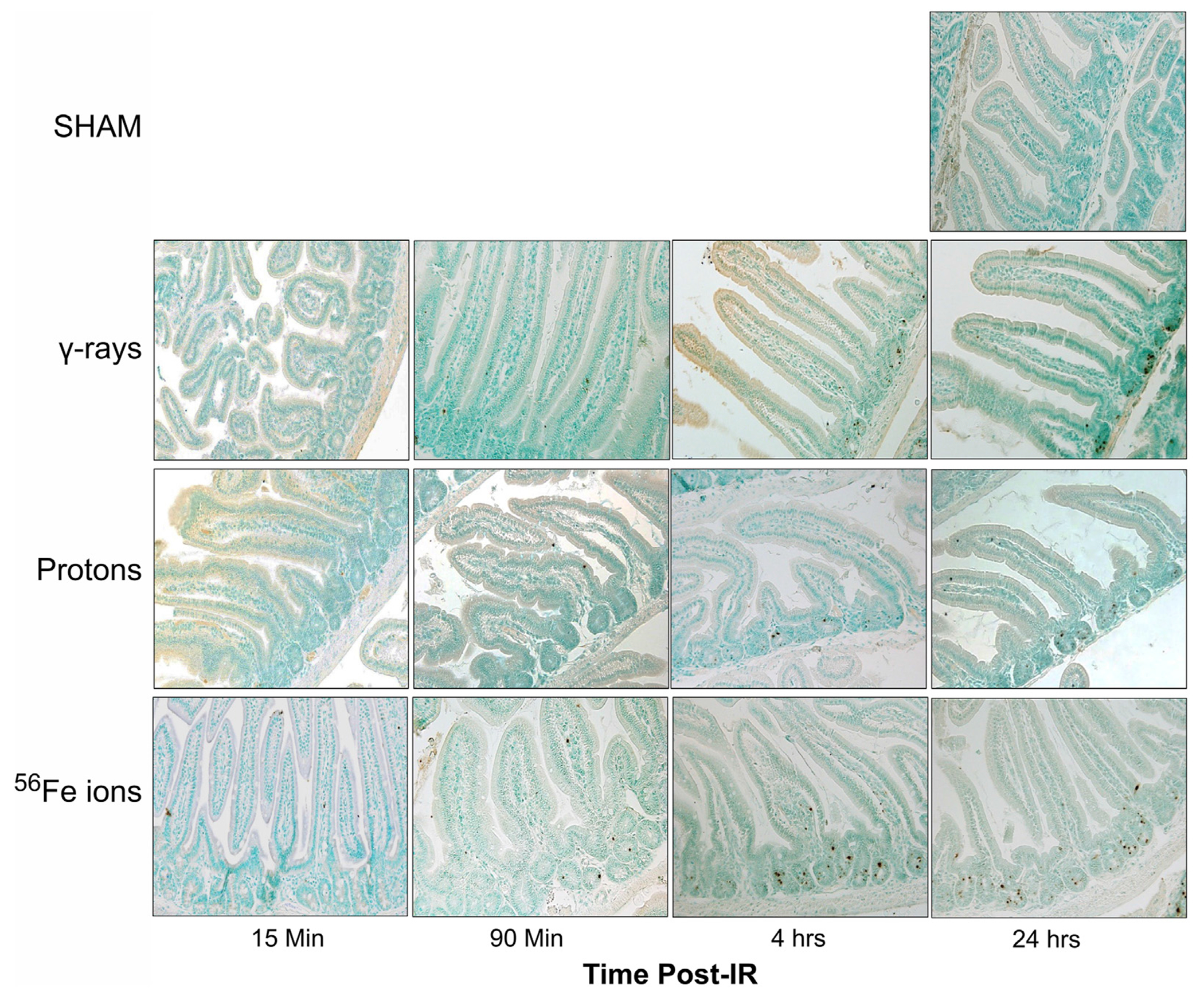
Visualization and quantitation of IR-induced apoptosis via TUNEL assay. Representative sections of duodenum collected (at various time points post-IR) from mice exposed to sham (0 cGy), 100 cGy of cesium-137 γ-rays, 100 cGy of 50 MeV protons, or 50 cGy of 1 GeV/n Fe-56 ions stained by terminal deoxynucleotidyl transferase dUTP nick end-labeling (TUNEL) to detect apoptotic DNA fragmentation (×20 magnification; 50-μm scale bar).

**FIGURE 9:**
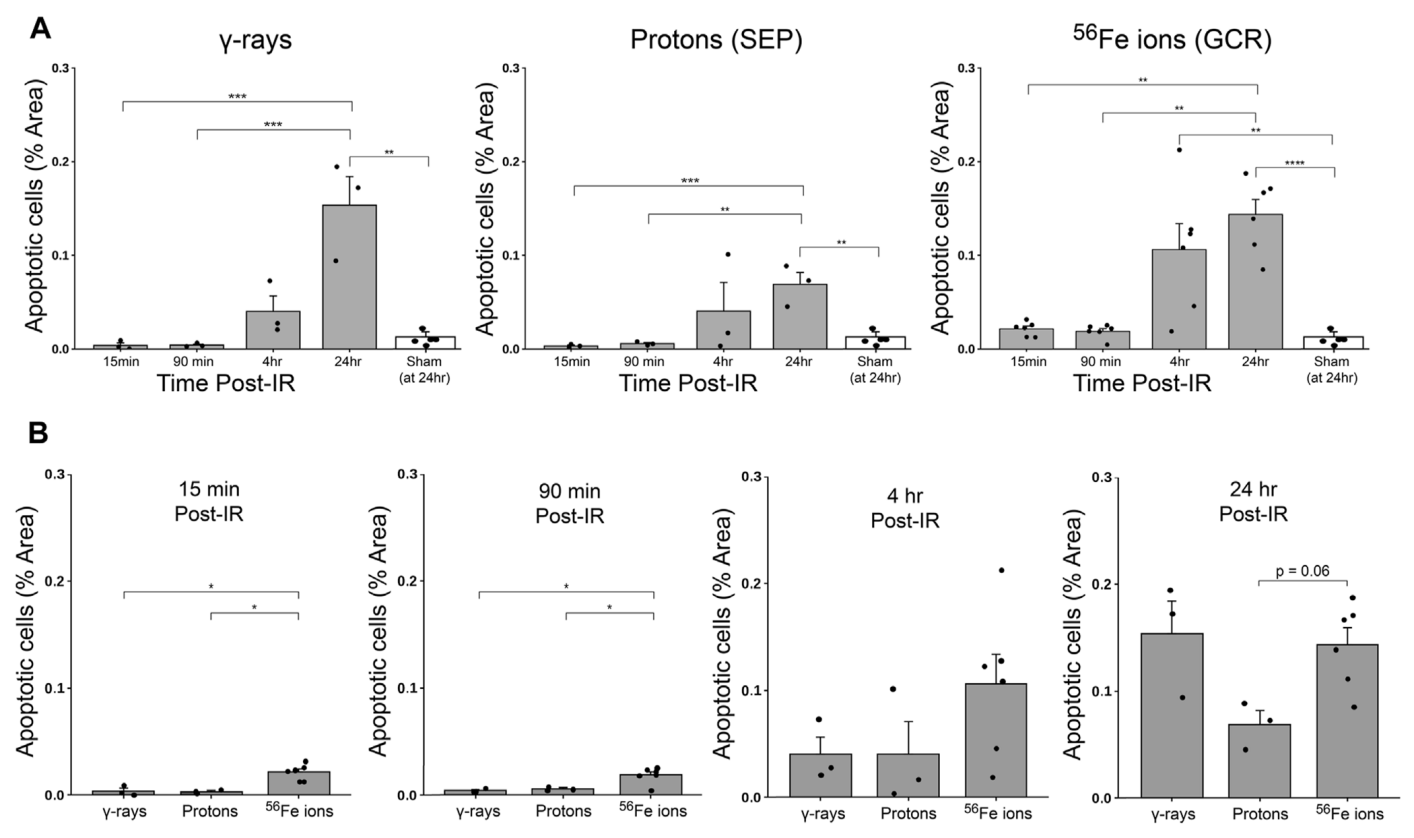
Quantification of apoptotic cells within duodenal sections from mice exposed to various IR schemes, using a custom ImageJ script;data presented as a function of IR type in panel **(A)** and by time post-IR in panel **(B)** (*n* = 3–6 depending upon IR scheme); *p < 0.05; **p < 0.01; ***p < 0.001; ****p < 0.0001.

To further examine GI effects of modeled SEP/GCR radiation *in vivo*, TUNEL assays were performed to assess apoptosis induction in response to the different IR types from 15 min to 24 h. All IR types increased levels of TUNEL-positive cells with signal detectable in some samples at 15 min post-IR and steadily increasing over time (representative images shown in Figure 8). To quantitate these levels, we again developed and applied a custom ImageJ script to analyze three regions of interest (ROIs) per mouse. Apoptotic cells in each ROI were quantified as the percent of the area that were TUNEL-positive using the equation: [DAB/Negative Signal * 100]. Each ROI was filtered to remove unwanted artifacts, and size, circulation, and color (DAB/Brown) were then used to identify positive DAB signals. Comparing these sections, we see that exposure to 50 cGy of Fe-56 ions led to a significantly higher TUNEL signal compared to 100 cGy of either protons or cesium-137 γ-rays at both the 15- and 90-min timepoints. Furthermore, the Fe-56 ion-irradiated group had a significantly higher TUNEL signal at both the 4- and 24-h time points compared to protons. Figure 9A shows the quantitative data obtained with the ImageJ script per IR type while Figure 9B presents these same data arranged per timepoint. When analyzing these images, we noted apoptosispositive cells were typically present at the base of intestinal crypts (notably in mice exposed to the iron ions) indicating that IR exposures resulted in ISC apoptosis (Figure 9A).

### Quantification of DNA double-strand breaks (DSBs)

**FIGURE 10:**
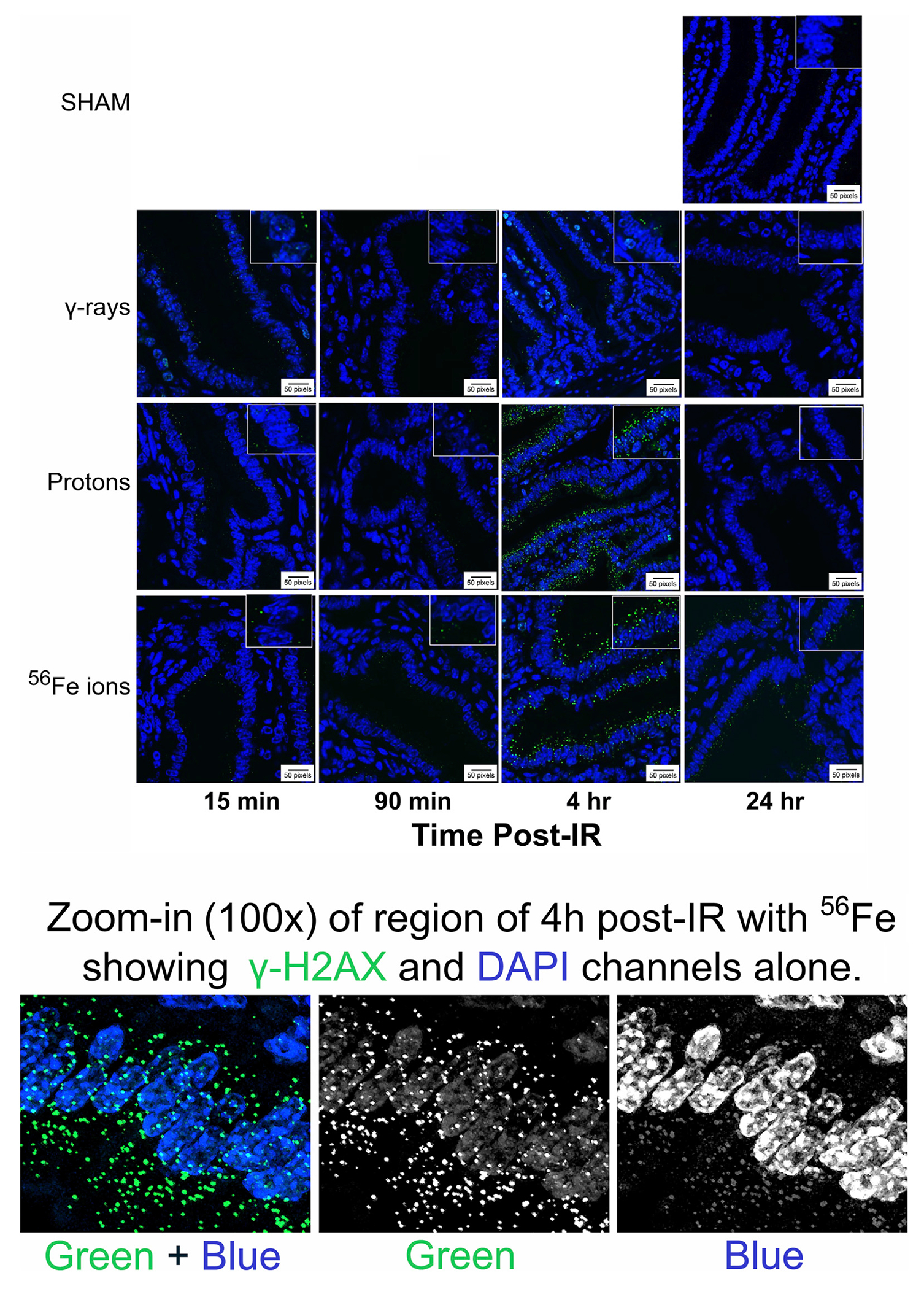
Visualization and quantitation of IR-induced DNA double-strand break (DSB)-associated nuclear foci. Panel A) Representative sections of duodenum collected (at various time points post-IR) from mice exposed to sham (0 cGy), 100 cGy of cesium-137 γ-rays, 100 cGy of 50 MeV protons, or 50 cGy of 1 GeV/n Fe-56 ions stained with an antibody specific to γ-H2AX pS139 (green) as a marker of IR-induced DSBs (×40 magnification; 20-μm scale bar). The panel at the bottom of the figure shows a high magnification of the area in the red-dashed region, with the individual γ-H2AX (green) and DAPI (blue) channels shown. DAPI (blue) staining of these cytoplasmic γ-H2AX (green) foci confirms that they are composed of DNA fragments.

**FIGURE 11:**
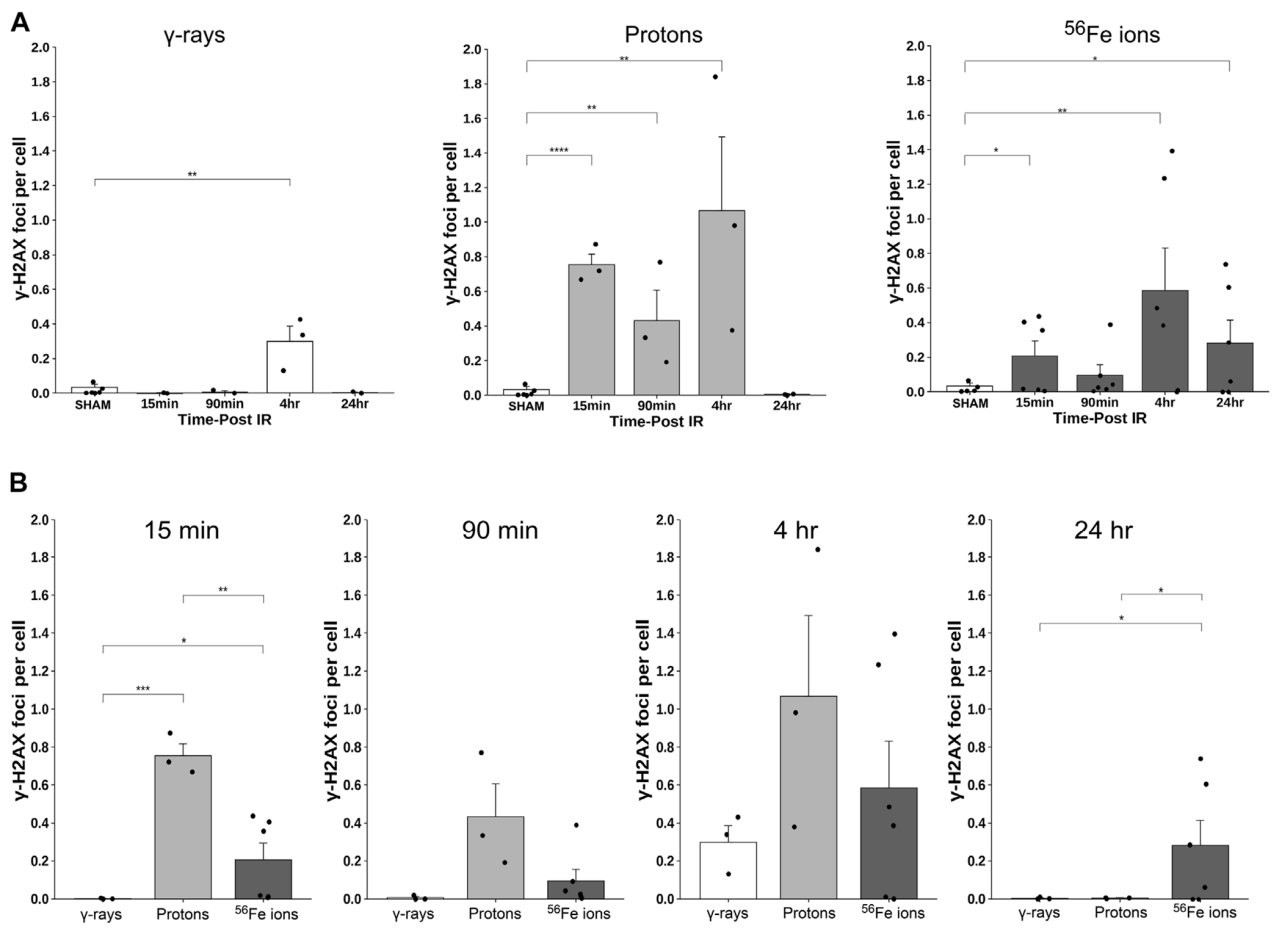
Quantification of γ-H2AX foci using a custom ImageJ script; data presented as a function of IR type in panel **(A)** and by time post-IR in panel **(B)** (*n* = 3–5 depending upon IR scheme); *p < 0.05; **p < 0.01; ***p < 0.001; ****p < 0.0001.

To assess the induction and resolution of DNA double-strand break (DSB)-associated nuclear foci resulting from exposure to each IR scheme, we again utilized a custom ImageJ script to quantify intranuclear γ-H2AX pS139 foci/cell in 3 ROIs from each mouse intestinal section. These analyses showed increased foci levels at both 15 min and 4 h post-IR with levels returning to baseline (sham-irradiated control levels) by 24 h post-IR in all of the mice treated with γ-rays or protons. In contrast, the number of γ-H2AX foci were still significantly higher than spontaneous background levels in the duodenum of mice exposed to Fe-56 ions, indicating that DSBs induced by these HZE ions were not fully repaired by this time—consistent with previous reports (Hada and Sutherland, 2006; Asaithamby et al., 2008; Wilson et al., 2010; Mladenov et al., 2018; Bennett et al., 2022). What was more striking was the observation of prominent extranuclear, cytoplasmic DNA fragments that were both DAPI-positive (i.e., DNA-containing) and gamma-H2AX pS139-positive (i.e., containing at least nucleosome-level chromatin) in the charged particle-irradiated samples only. Representative confocal images of γ-H2AX staining appear in Figure 10 and demonstrates the strong intensity and location of these brightly staining cytosolic DNA fragments. The panel at the bottom of Figure 10 shows a high magnification of the area in the red-dashed region, with the individual γ-H2AX (green) and DAPI (blue) channels shown. DAPI (blue) staining of these cytoplasmic γ-H2AX (green) foci confirms that they are composed of DNA fragments. Figure 11A shows a graphical presentation of the quantitative data obtained with the custom ImageJ script, with each panel showing the intranuclear γ-H2AX signal as a result of each IR species; Figure 11B presents these data per time post-IR. It remains to be determined in future studies if the cytoplasmic DNA fragments resulted from charged particle-induced chromothripsis, or chromosomal shattering (Mladenov et al., 2018), with the subsequent egress of small DNA fragments through the damaged/leaky nuclear membrane into the cytosol, and whether this results in activation of cGAS/STING signaling pathways and/or TREX1-mediated degradation of these fragments (Yang et al., 2007; Vanpouille-Box et al., 2017; Durante and Formenti, 2018) given that they are no longer present at 24 h in any of the irradiated samples.

### Testing curcumin-laden nanolipoprotein particles (cNLPs) as a GI-specific MCM for SEP/GCR radiation exposures

Our other ongoing functional and multi-omic analyses of space radiation and microgravity effects on human and mouse *in vitro* and *in vivo* model systems have yielded comprehensive datasets on molecular signaling pathways involved in SEP/GCR effects on human hematopoiesis and leukemogenesis and provided targets for developing targeted MCMs (Rodman et al., 2017; Almeida-Porada et al., 2018; Low et al., 2018). These analyses have identified multiple affected signaling cascades that can potentially be modulated by curcumin, a natural bioactive compound derived from turmeric (Curcuma longa) that has been shown to have anti-proliferative and pro-apoptotic effects in a variety of cancer models. Curcumin has also been reported to serve as both a radiosensitizer for cycling cell populations (e.g., tumor cells) and a radioprotector in non-cycling, quiescent cell populations/tissues through its potent antioxidant activities (Sebastia et al., 2014). To overcome curcumin’s inherently poor bioavailability, we recently reported on the successful development and characterization of ApoA1-based nanolipoprotein particles (NLPs) loaded with high levels of curcumin (cNLPs) and show this NLP platform markedly increases curcumin’s solubility and allows us to lyophilize them for long-term storage and use via injection or oral routes. We also showed that pretreatment of primary human fibroblasts with cNLPs provided significant radioprotection when administered 18–24 h pre-irradiation and radiomitigation when administered 15 min post-irradiation, reduced γ-H2AX-positive DSB-associated nuclear foci, and modulated expression of genes involved in the DNA damage response (DDR) following cesium-137 γ-ray irradiation (Evans et al., 2022). In this study, we sought to test whether these cNLPs could serve as a GI-specific MCM against the damaging effects of SEP/GCR radiation *in vivo*. We therefore pretreated C57BL/6J mice with 27 μM (10 μg/mL) cNLPs (the optimum radioprotective concentration derived in our *in vitro* experiments) 18–24 h prior to exposure to 100 cGy of γ-rays or protons, or 50 cGy of Fe-56 ions, and assayed the duodenal samples from the cNLP cohorts identically as described above.

**FIGURE 12:**
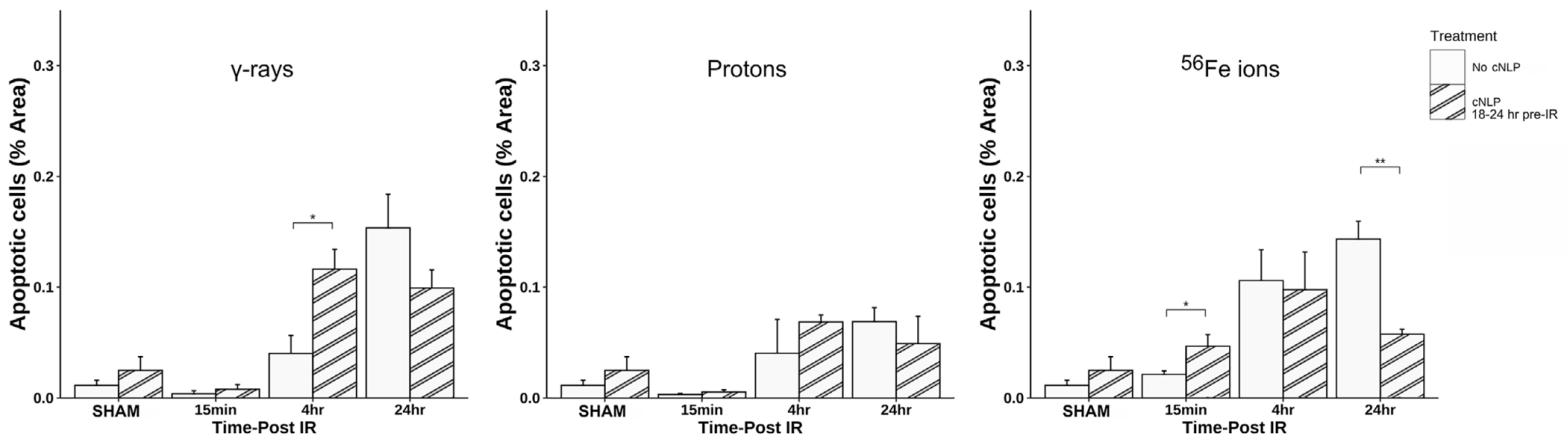
Efficacy of cNLPs as a candidate GI MCM for SEP/GCR radiation. Quantification of apoptotic cells via TUNEL assay as above using a custom ImageJ script; data presented as a function of IR type in each panel. Open bars are data from mice that did not receive cNLPs; striped bars are data from mice that received 27 μM cNLPs via IV tail-vein injection 18–24 h pre-IR (*n* = 3–6 mice depending upon IR scheme); *p < 0.05; **p < 0.01.

**FIGURE 13:**
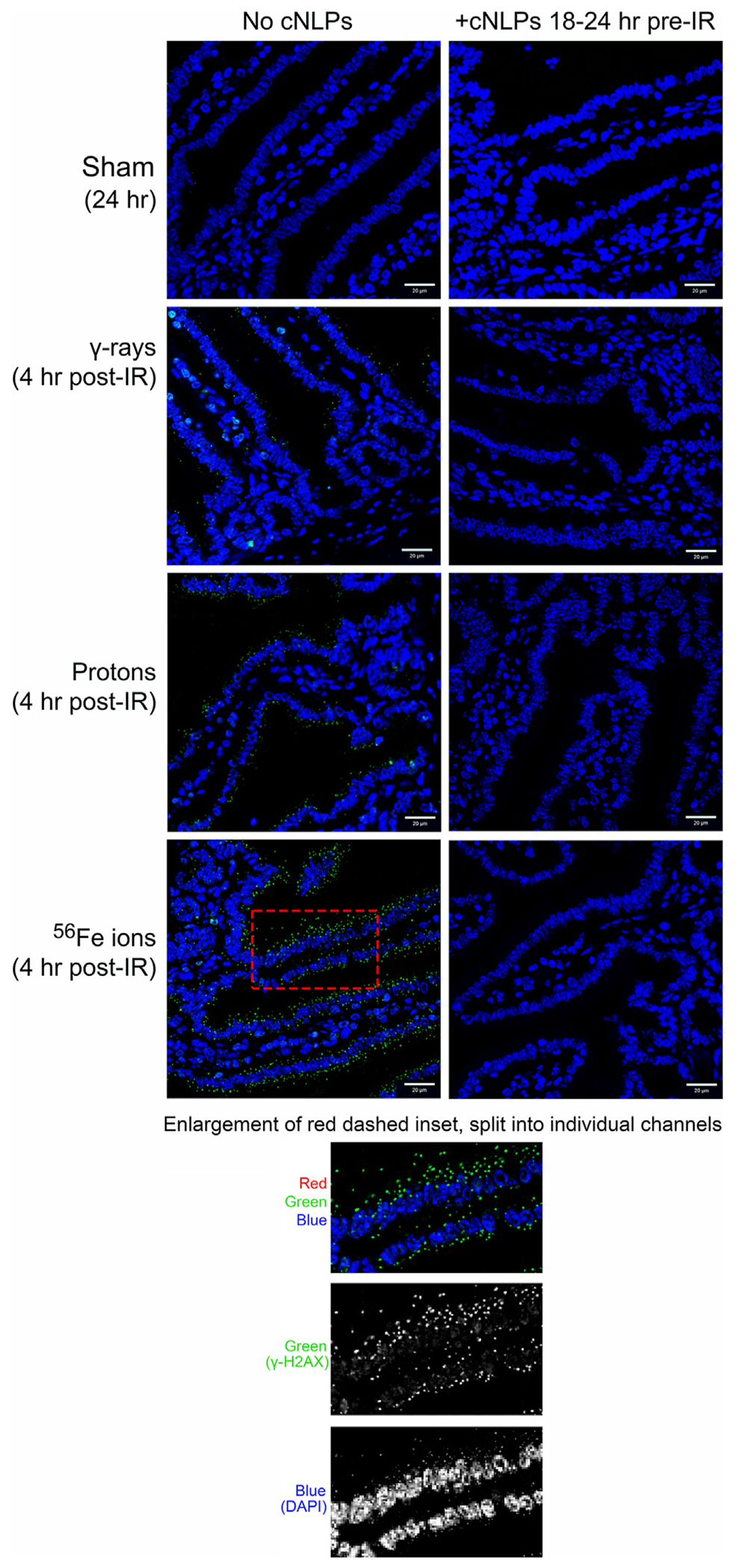
Efficacy of cNLPs as a candidate GI MCM for SEP/GCR radiation. Representative sections of duodenum collected 4 h post-irradiation following 100 cGy of cesium-137 γ-rays, 100 cGy of 50 MeV protons, or 50 cGy of 1 GeV/n Fe-56 ions and sham controls collected at 24 h time point stained with a γ-H2AX pS139-specific antibody (green) to detect DSB-associated nuclear foci. Left column shows samples from mice that did not receive cNLPs; right columns are samples from mice that received cNLPs (×40 magnification; 20-μm scale bar). The panel at the bottom of the figure shows a higher magnification (×100) of an area from the duodenum of a Fe-56 ion-exposed mouse collected at 4 h post-IR, with individual γ-H2AX (green) and DAPI (blue) channels shown, confirming they are DNA fragments.

**FIGURE 14:**
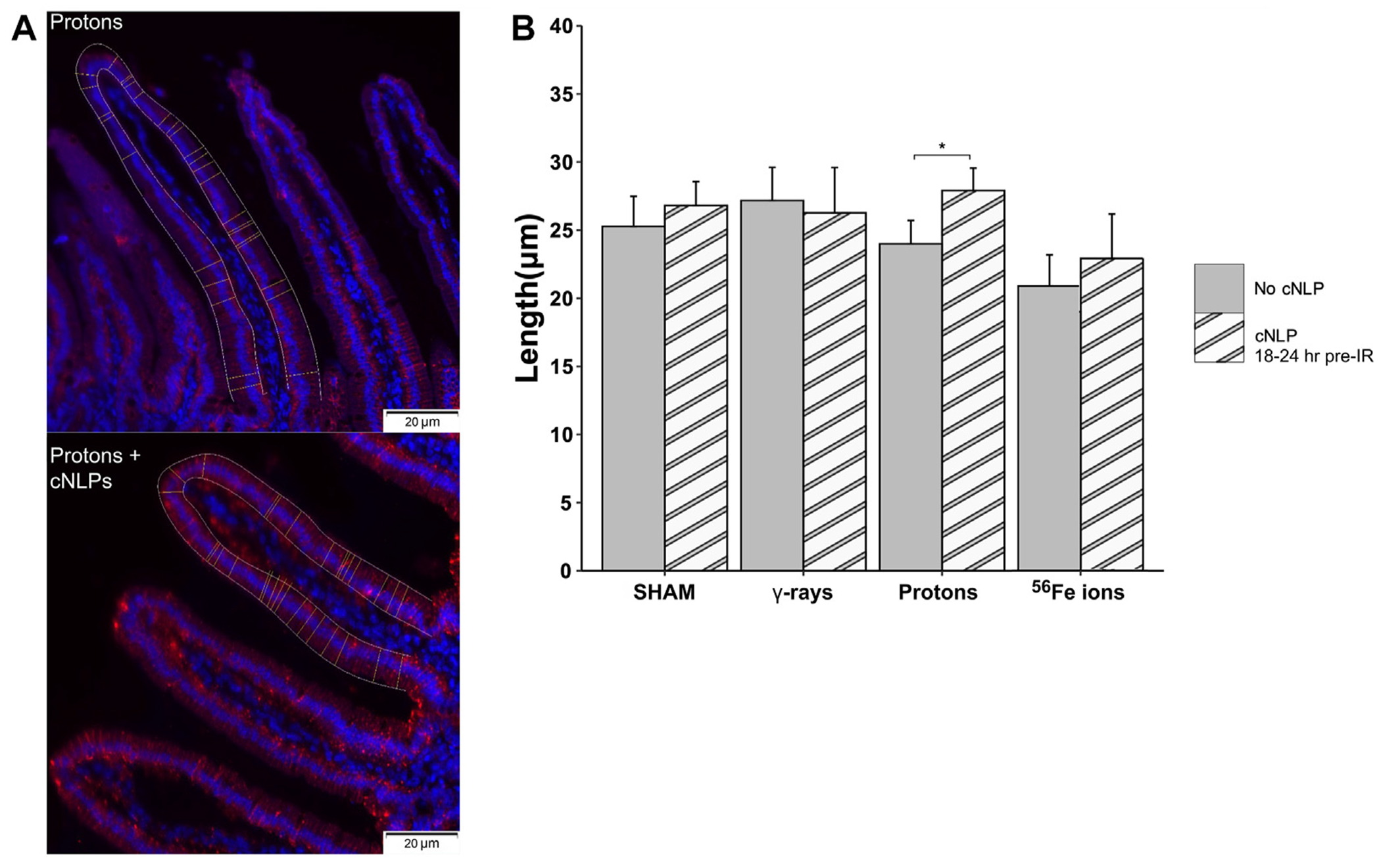
Ability of cNLPs to restore epithelial barrier function. Panel **(A)** Representative sections of duodenum collected 24 h post-IR from mice exposed to 50 MeV protons without (top panel) or with cNLP pretreatment (bottom panel) stained with an antibody to tight junction-associated Claudin-3 (red, ×40 magnification; 20-μm scale bars). Panel **(B)** Quantification of Claudin-3 barrier length assessed by custom ImageJ script; data presented as a function of IR type (*n* = 3–6 depending upon IR scheme); *p < 0.05.

As can be seen in Figure 12, 27 μM cNLP pre-treatment via IV injection reduced the incidence of apoptosis assessed by TUNEL staining using the previously described custom ImageJ script in mice exposed to all 3 radiation types 24 h post-IR, but this decrease only achieved statistical significance in the group exposed to Fe-56 ions. Similarly, radioprotective effects were also seen when we analyzed the incidence of DSB-associated foci via γ-H2AX pS139 staining. As shown in Figure 13, cNLP treatment reduced the number of γ-H2AX foci-positive cells in the duodenum resulting from exposure to γ-rays, protons, and Fe-56 ions at 4 h post-IR (the point of maximal DSB induction in these studies), as well as nearly eliminated the appearance of the gamma-H2AX-positive cytosolic DNA fragments. The panel at the bottom of Figure 13 shows a high magnification of the area in the red-dashed region, with the individual γ-H2AX (green) and DAPI (blue) channels shown. DAPI (blue) staining of these cytoplasmic γ-H2AX (green) foci confirms that they are composed of DNA fragments. Confocal immunofluorescence studies further demonstrated an increase in Claudin 3 staining (Figure 14A) using our ImageJ script, revealing an improvement in the lengths of the epithelial tight junctions in both the proton- and Fe-56 ion-exposed groups. This difference, however, only achieved statistical significance in the proton-exposed group (Figure 14B).

## Discussion

It is well appreciated the GI system constitutes one of the most radiosensitive tissues of the body vulnerable to both the direct (DNA damaging) and indirect (inflammation-associated) effects of IR exposure due to the rapid cellular turnover needed to continually replenish the epithelial layer of the villi within the small intestine as the epithelial cells at the villi tips are sloughed off as part of normal GI function/wear-and-tear. This process of epithelial replenishment is driven by a small population of intestinal stem cells (ISC) that reside within the crypts at the base of the villi. Even relatively low dose IR exposures can induce crypt cell apoptosis and mucosal lesions, leading to loss of epithelial barrier function and increased mucosal permeability. This increased permeability induces nutrient and fluid loss and allows gut pathogen infiltration. The net effect of these insults is the establishment of a state of chronic mucosal inflammation, which results in tissue loss, fibrosis, and decreased motility (Shadad et al., 2013; Shim et al., 2015; Lee et al., 2019; Chen et al., 2021). With extended deep space missions beyond LEO exposing astronauts to both chronic LDR and sporadic acute HDR charged particles, the higher RBE values for GI-related pathologies documented following low doses exposures presents a major concern for NASA’s risk assessment and challenge to astronaut radioprotection and mitigation strategies.

IR is an established risk factor for the development of colorectal cancer (CRC) (Thompson et al., 1994; Pawel et al., 2008; Grant et al., 2017), and NASA-funded studies performed by the Fornace and Datta group demonstrate exposures to low doses (10 and 50 cGy) of protons and 1 GeV/n Fe-56 ions significantly enhances the development and progression of intestinal tumors in mice harboring mutations in the Apc gene due to both direct DNA damage and via increased production of reactive oxygen species (ROS) (Trani et al., 2014; Suman et al., 2016; Suman et al., 2017; Kumar et al., 2018; Kumar et al., 2019; Suman et al., 2020; Suman et al., 2021). The persistent DNA damage resulting from HZE exposures also caused premature senescence and emergence of a senescence-associated secretory phenotype (SASP) within the ISC pool of mice exposed to 50 cGy of Fe-56 ions (Kumar et al., 2018; Kumar et al., 2019), and a recent study from this group highlights the majority impact that exposures to HZE ion components of simulated mixed-field exposures have on GI tumorigenesis (Suman et al., 2022). These studies have primarily focused on the longer-term effects of such exposures, while fewer studies have explored acute/immediate effects of irradiation within the first 24 h post-irradiation (Purgason et al., 2018), a time period in which intervention with an appropriate GI-specific MCM may have the most beneficial effects, especially when considering potential higher dose/dose-rate exposures that may occur if astronauts are exposed to a SPE.

In the present report, we describe the results of ground-based studies performed at NSRL to address these early timepoints in C57BL/6J mice exposed to doses of modeled SEP/GCR radiation designed to approximate the total dose an astronaut will receive during a 3-year Mars mission. Due to time, funding, and facility access/cost restraints, we were limited to conducting acute HDR irradiations and could not explore potential dose-rate effects that may occur if the exposures were extended over several weeks, months or years, though we agree dose-rate is a very critical factor to consider in future studies (Rithidech et al., 2013; Bennett et al., 2022). The mice in this study were euthanized at time points ranging from 15 min to 24 h post-irradiation, with their small intestines collected to characterize GI-related effects and test the ability of curcumin-laden nanolipoprotein particles (cNLPs) we recently developed and characterized (Evans et al., 2022) to serve as a much-needed MCM to protect the GI tract of astronauts from the damaging effects of SEP/GCR radiation.

Our results show that both SEP and GCR radiation induces immediate histological and functional alterations within the GI tract of C57BL/6J mice, disrupting epithelial tight junctions and triggering an inflammatory response, leading to dramatic enlargement/swelling of the lymph nodes/MALT (mucosa-associated lymphoid tissue/Payer’s patches)—a finding that was unique to some mice exposed to protons and Fe-56 ions. The findings of these alterations in the lymphoid tissue only in mice exposed to protons and Fe-56 ions is likely a result of the differing effects charged particle radiations have been reported to exert on the immune system (Gridley et al., 2002; Schmid and Multhoff, 2012; Hoehn et al., 2019). Exposure to Fe-56 ions also led to marked vascular congestion and edema within the villi in some of the mice, as well as a trend towards a decrease in villi height and evidence suggestive of possible epithelial loss. An important caveat to our findings is that these alterations were only seen in some of the tissue sections from specific mice within the Fe-56 ion-exposed group, which may be the result of mouse-to-mouse variations in handling, diet, etc.

Our conclusions regarding disruption of the intestinal epithelial barrier were based upon quantitative measures of duodenal sections stained with an antibody specific to Claudin-3 and analyzed with a custom script in ImageJ to measure junction lengths between adjacent epithelial cells in an unbiased manner. If SEP/GCR-induced reduction in Claudin-3 junction length translates into epithelial disruption, such exposures would be predicted to lead to increased gut barrier permeability, i.e., leaky gut, which can allow passage of bacteria and toxins into the bloodstream (Fukui, 2016; Kumagai et al., 2018), producing symptoms including fatigue, joint pain, rashes, and food sensitivity and placing the immune system in a hyperactivated state. Astronauts on long duration missions may thus be at risk of chronic digestive issues, e.g., bloating, gas, diarrhea, constipation, and food sensitives/intolerance (Camilleri, 2019) as a result of SEP/GCR-induced damage to their GI tract. We also evaluated the effects of SEP/GCR and how exposure can disrupt tissue homeostasis by depleting intestinal stem cells (ISC), the pool of which is maintained by a delicate balance between cell division and cell death/turnover (Prise and Saran, 2011). While quantitative analyses using antibodies to PCNA and the ISC marker Msi-1 (Potten et al., 2003) did not reveal statistically significant changes in the rate of ISC proliferation, qualitative changes were readily evident based upon visual evaluation of some of the sections by confocal microscopy, with a trend towards an increase in proliferating cells localized within the crypts as a result of IR exposure. Additional studies are underway to examine this issue in more detail, as IR-induced DNA damage to the ISC, combined with enhanced proliferation could lead to the propagation of ISC harboring mutations that could lead to tumor development within the GI tract. Further studies are also planned using machine learning (ML) approaches to examine the duodenal sections at higher resolution to answer the important question of whether SEP/GCR exposure leads to alterations in ISC positioning within the crypt/villi.

Apoptosis/programmed cell death is a normal part of GI homeostasis that ensures aged/damaged cells are safely and efficiently removed and replaced to maintain tissue function (Wan et al., 2022). However, excessive apoptosis within the villi can lead to villous atrophy (Schuller et al., 2006; Hauer-Jensen et al., 2014), as occurs in celiac disease, and can lead to erosion and blunting of the villous tips, resulting in malabsorption of nutrients, diarrhea, and weight loss (Potten, 2004; Williams et al., 2010; Martins et al., 2016). To assess the presence and incidence of IR-induced apoptosis within the duodenum of mice exposed to γ-rays, protons, or Fe-56 ions, quantitative analysis of duodenal sections stained with a chromogenic TUNEL assay and a custom ImageJ script. These analyses demonstrated that mice exposed to a 50 cGy dose of Fe-56 ions had greater TUNEL signal at 4 h post-exposure compared to 1 Gy of γ-rays, reflecting the higher relative biological effectiveness of these HZE ions. Visual inspection of TUNEL-stained duodenal sections revealed the presence of TUNEL-positive cells within the crypts where ISC reside, in agreement with prior studies with mice exposed to high doses (15 Gy) of γ-rays (Hornsey, 1973; Potten, 2004).

One of the primary mechanisms by which IR, especially high LET HZE ions, causes cellular/tissue injury and triggers apoptosis is via DNA double-strand breaks (DSB) induction, can lead to chromosomal aberrations and mitotic cell death and/or apoptosis depending on cell/tissue type. Due to considerations of the track structure and energy deposition patterns of both protons and HZE ions, they are more effective per unit dose than gamma-ray-induced photoelectrons at generating higher levels as well as denser clustering of DSBs and other oxidative base lesions that is more difficult for the cellular DDR machinery to recognize and repair (Sutherland et al., 2001; Hada and Sutherland, 2006; Asaithamby et al., 2008; Mladenov et al., 2018). Phosphorylation of the histone variant H2AX on serine 139 to generate γ-H2AX plays a key role in rapidly recruiting DDR signaling repair proteins to DSB sites (Rogakou et al., 1999; Paull et al., 2000) which are typically resolved back to spontaneous background levels by 24 h post-irradiation in most normal human and rodent cell lines/strains following low LET photon and proton irradiation, with higher levels of residual foci above background levels observed after exposures intermediate to high LET HZE ions (Saha et al., 2014; Bennett et al., 2022). We observed intranuclear γ-H2AX foci post-irradiation in all IR groups with peak γ-H2AX focus formation observed 4 h post-IR.

Surprisingly, our studies also demonstrated the accumulation of cytoplasmic DAPI-positive and γ-H2AX-positive signals in the cytosol of cells within duodenal samples from mice exposed to protons and Fe-56 ions only, suggesting that this response may be specific to charged particle irradiations. Whether these charged particles have the ability to trigger chromothripsis (“shatter” chromosomes (Mladenov et al., 2018)), with subsequent leakage of small DNA fragments through the damaged nuclear membrane is an important observation that will require additional studies. Cytoplasmic DNA has been shown to activate cGAS/STING-mediated extranuclear DNA sensing/signaling pathways (Durante and Formenti, 2018) and activate TREX1-dependent cytosolic DNA cleavage. These may become important candidate pathways to potentially target for charged particle-specific MCMs that would find application for both space radiation and hadron radiotherapy-associated normal tissue radioprotection. It is also important to consider the microdosimetry of these exposures as related to their relative fluences in the deep space and/or Mars/lunar radiation environments. For the IR types and doses used in these studies, the mean numbers of particle traversals per cell nucleus equates to 265 protons/nucleus for the 100 cGy dose of 50 MeV protons compared to only ~1.1 iron ions/nucleus for 50 cGy dose of 1 GeV/n Fe-56 ions, assuming a mean nuclear surface area of ~50 μm2 (determined from our image analyses).

In addition to characterizing responses of the murine GI system to SEP/GCR radiation, we also performed studied to assess the ability of our candidate curcumin-based nanoparticle MCM (Evans et al., 2022)—cNLPs—to provide GI radioprotection for low to intermediate dose SEP/GCR exposures (including those typical of a SPE), based upon the promising results we recently reported with this MCM in human fibroblasts (Evans et al., 2022). We tested the efficacy of cNLPs when administered intravenously 18–24 h prior to IR exposure in this *in vivo* mouse model to ascertain whether such pretreatment could provide radioprotection from SEP/GCR to the GI system *in vivo*. Our data on IR-induced levels of apoptosis, DSB-associated nuclear foci induction, and epithelial barrier integrity by Claudin-3 staining provides compelling evidence that cNLPs afforded a promising degree of radioprotection in the normal murine small intestine, reducing the incidence of apoptosis and diminishing γ-H2AX foci levels at 4 h post-exposure to protons and to Fe-56 ions. Pretreatment with cNLPs also led to an increase in Claudin-3 signal and some lengthening of the tight junctions between neighboring epithelial cells within the villi of the duodenum was observed, but this effect only achieved statistical significance in the group exposed to protons. We expect that the radioprotective effects of the cNLPs are being mediated, at least in part, by curcumin’s established ability to decrease ROS/RNS production by decreasing inducible nitric oxide synthase (iNOS), increasing glutathione peroxidase, as well as increasing the transcription of antioxidant gene response elements through the Nrf2-Keap1 pathway (Aggarwal et al., 2007).

In conclusion, these studies have identified immediate changes in the GI tract caused by low to intermediate dose SEP/GCR-relevant charged particle exposures, giving us a better understanding of potential acute space radiation-induced physiological and molecular changes within the GI system which are relevant when considering the potential for a higher dose/dose-rate SPE during extended missions. With further research and optimization, we envision the cNLPs we have tested herein (and the oral and liquid formulations we have since developed) will yield a viable MCM and contribute to a successful radioprotection strategy that can promote astronaut safety and mission success during prolonged deep space explorations beyond LEO.

## Data Availability

The original contributions presented in the study are included in the article/supplementary material, further inquiries can be directed to the corresponding author.
